# Homeopathic Doses of *Gelsemium sempervirens* Improve the Behavior of Mice in Response to Novel Environments

**DOI:** 10.1093/ecam/nep139

**Published:** 2011-02-15

**Authors:** Paolo Bellavite, Paolo Magnani, Elisabetta Zanolin, Anita Conforti

**Affiliations:** ^1^Department of Morphological Biomedical Sciences (Chemistry and Microscopy Section), University of Verona, Verona 37134, Italy; ^2^Department of Medicine and Public Health (Biomedical Statistics Section), University of Verona, Verona, Italy; ^3^Department of Medicine and Public Health (Medical Pharmacology Section), University of Verona, Verona, Italy

## Abstract

*Gelsemium sempervirens* is used in homeopathy for treating patients with anxiety related symptoms, however there have been few experimental studies evaluating its pharmacological activity. We have investigated the effects of homeopathic doses of *G. sempervirens* on mice, using validated behavioral models. Centesimal (CH) dilutions/dynamizations of *G. sempervirens*, the reference drug diazepam (1 mg/kg body weight) or a placebo (solvent vehicle) were intraperitoneally delivered to groups of mice of CD1 strain during 8 days, then the effects were assessed by the Light-Dark (LD) choice test and by the Open-Field (OF) exploration test, in a fully blind manner. In the LD test, the mean time spent in the illuminated area by control and placebo-treated animals was 15.98%, for mice treated with diazepam it increased to 19.91% (*P* = .047), while with *G*. *sempervirens* 5 CH it was 18.11% (*P* = .341, non-significant). The number of transitions between the two compartments increased with diazepam from 6.19 to 9.64 (*P* < .001) but not with *G*. *Sempervirens*. In the OF test, *G*. *sempervirens* 5 CH significantly increased the time spent and the distance traveled in the central zone (*P* = .009 and *P* = .003, resp.), while diazepam had no effect on these OF test parameters. In a subsequent series of experiments, *G*. *sempervirens* 7 and 30 CH also significantly improved the behavioral responses of mice in the OF test (*P* < .01 for all tested variables). Neither dilutions of *G*. *sempervirens* affected the total distance traveled, indicating that the behavioral effect was not due to unspecific changes in locomotor activity. In conclusion, homeopathic doses of *G*. *sempervirens* influence the emotional responses of mice to novel environments, suggesting an improvement in exploratory behavior and a diminution of thigmotaxis or neophobia.

## 1. Introduction

In homeopathic *Materia Medica*, *Gelsemium sempervirens* (Loganaceae) is described as a remedy for a variety of neurological and behavioral symptoms including general prostration, drowsiness, tiredness, mental apathy, lack of muscular coordination and discomfort when confronted with novelty or unfamiliar situations; these symptoms are alleviated by motion and aggravated by emotion and excitement [1–3]. However there have been only a few experimental studies supporting this assertion [4]. Therefore, there is scope for testing this compound in homeopathic dilutions on validated behavioral models of emotional state and anxiety. *G. sempervirens* is a twining vine native to warm temperate and tropical America, from Guatemala north to the southeastern USA. All parts of this plant contain the toxic strychnine-related alkaloids gelsemine, gelseminine and sempervirine [5]. Neurological signs characterized by marked progressive weakness and convulsions culminating in death have been observed in goats after ingestion of *G*. *sempervirens* and ensuing toxicosis [6]. At pharmacological doses, *G*. *sempervirens* has been reported to show sedative, analgesic and anti-seizure properties [7–9]. In an early report [10], ultra-low doses of *G*. *sempervirens* in mice were found to counter the behavioral effects of the anxiogenic compound RO 15-3505 (inverse agonist of benzodiazepines). More recently, Bousta et al. report that in some but not all experimental conditions, homeopathic dilutions of *G*. *sempervirens* reduce stress-induced behavioral alterations of mice in the staircase and light-dark (LD) tests [11]. However, these results consist of reversing the effects of severe stress (conditioned paradigm), and the findings vary widely depending on the dose administered and test performed.

In this article we report a series of experiments testing the possible effects on mouse behavioral responses to *G*. *sempervirens* in homeopathic dilutions/dynamizations. Two validated tests on animal models, namely the LD choice test and the open-field (OF) test, were used in order to acquire various behavioral parameters widely used in neuropsychopharmacology for drug screening [12–14]. We performed the study on the unconditioned responses, using ethologically based paradigms which involve the spontaneous reactions to non-painful stimuli. The LD test is based on the innate aversion of rodents to brightly lit area: control mice placed in the large, bright, section will rapidly move into the dark chamber and a classic anxiolytic effect manifests itself as an increase in the percentage of time spent in the light area and in the number of transitions between the two compartments [15]. The OF test involves placing an animal in an unknown environment in order to observe a number of behavior patterns. The tendency to stay on the periphery of the field is known as thigmotaxis and is often considered an index of highly emotional behavior [16, 17]. Conversely, the percentage of time spent in the central zone and the distance traveled in the central zone are considered indicative of exploratory behavior and should reflect a decrease in anxiety.

In homeopathy, a wide range of dosages are used. Such formulations are better described as homeopathic dilutions (also called “dynamizations”, or “potencies”), whose preparation procedure involves successive steps of dilution—10× or Decimal (D), 100× or Centesimal (CH)—followed by strong manual or mechanical succussion. Homeopathic dilutions/dynamizations such as 5 CH are expected to contain ultra-low concentrations of the purported active ingredient, but in amounts still within the molecular range since the dilution factor from the first ethanolic extract (mother tincture) is 10^10^. In this work we chose to start the investigation with a series of experiments testing the effects of the 5 CH dilution/dynamization, which is currently used not only in homeopathic therapy but is also in the domain of “conventional” pharmacology and of current scientific wisdom. Since homeopathy also makes use of high dilutions/dinamizations, in an ensuing series of experiments, we investigated the effects of further dilutions/dynamizations, namely *G. sempervirens* 7 and 30 CH.

## 2. Methods

### 2.1. Subjects and Maintenance

Male 4-5-week-old mice of the specified inbred strains (CD1 or C57BL/J6) were purchased from Harlan Laboratories (Udine, I) and allowed to acclimate for 1 week before testing. Mice were randomly distributed 4/cage in standard polycarbonate cages (369 × 156 × 132 mm) with water and food available *ad libitum*, in an air-conditioned room (temperature 22±2°C, humidity *∼*55 ± 5%). Lights were left switched on between 7 a.m. and 7 p.m. Cages were cleaned and bottles were filled with fresh tap water thrice a week, excluding on the days of testing. The sequential order in which mice were injected and then tested was arranged so that the mean interval time between injection and testing was the same for all the experimental groups. *G*. *sempervirens* and the control vehicle (placebo) were administered intraperitoneally (i.p.) each day at 9 a.m. for 9 consecutive days, including on the 2 last days devoted to behavioral testing. Mice belonging to the benzodiazepine standard treatment group were also injected with the control solution for 9 days, but on the testing days the control solution was supplemented with diazepam (see below).

Apart from the drug injections and testing, the animals were not subjected to pain or other forms of emotional or physical stress. The animals were used only once in any given test to avoid the confounding effects of learning and habituation. After completing the tests, the mice were put back into their original home cages and then sacrificed using CO_2_-saturated atmosphere. All the tests were conducted at the Faculty of Medicine, Verona University, Italy, in accordance with the guidelines approved by the European Community Council Directive of November 24, 1986 (law86/609/CEE). The testing procedures were approved by the Animal Ethical Committee of the Verona University and by the Italian Health Ministry (authorization n. 4 Tit. VII/5, February 16, 2007).

### 2.2. Drugs

*Gelsemium sempervirens* in the 4, 6 and 29 CH dilutions/dynamizations was prepared by Boiron Laboratories (Lyon, F) from the mother tincture of plant roots, according to the French Pharmacopoeia [18] and supplied as a hydroalcoholic (30% ethanol, v/v) solution. The mother tincture was assayed to determine its gelsemine content, which proved to be 0.021%; since the molecular weight of this compound is 322.41, its concentration in the mother tincture used for preparing the homeopathic dilutions/dynamizations was 6.5 × 10^−4^ moles/l. The control solution (placebo) used in this investigation was provided by Boiron Laboratories, using the same hydroalcoholic solvent stock (30% ethanol, v/v) employed to prepare the *G*. *sempervirens* dilutions. On the morning of the first day of drug delivery, before starting the treatment, 0.4 ml samples of the solutions were added to 39.6 ml of distilled sterile and apyrogenic water in a sterile 50 ml Falcon plastic tube, closed with a plastic cap and the dilution was manually succussed with 20 strong vertical strokes to obtain the operative dilutions 5, 7 and 30 CH, with final ethanol concentration lowered to 0.3% (v/v). Since each dilution step involves a 100 × decrease in concentration, the “theoretical” concentrations of gelsemine in the 5, 7 and 30 CH solutions were 6.5 × 10^−14^, 6.5 × 10^−18^ and 6.5 × 10^−64^ moles/l, respectively. The operators who performed the injections and the behavioral tests were totally unaware (“blind”) of the treatment group to which the animals had been assigned. Blinding of all procedures of injection and testing was performed by randomizing and coding the tubes containing the drug and control solutions. The person who coded the solutions did not belong to the research group and signed a confidentiality agreement form; the codes were recorded on a sheet that was kept sealed inside an envelope until all the tests and calculations were completed. The coded solutions were distributed in 15 ml sterile Falcon plastic tubes (7.5 ml/tube), wrapped in aluminum foil and stored at +4°C until the day of use. Before administering the contents, each tube was manually shaken with 20 strokes. On the days of the behavioral tests, the mice were injected 60 min before the start of the tests, which were performed between 11 a.m. and 3 p.m. Each day, 0.3 ml of the remedy or control placebo were administered by i.p. injection using 1 ml insulin syringes. The benzodiazepine standard drug was diazepam (*Valium*, Roche) (5 mg/ml), diluted by an independent investigator to ensure blinding of operators with respect to group assignment, in the same control solution and administered intraperitoneally at the final dose of 1 mg/kg body weight (b.w.).

### 2.3. Behavior Assessment

A video-tracking camera (GZ-MG135, JVC, Japan) and a software program (“Smart” VTS system from PanLab, Barcelona, E) were used to record the sessions automatically. The camera viewed four test arenas, each one of which, in turn, was divided by the software into two different zones according to the test to be performed (LD or OF). All the sessions were recorded and stored as DVD. The video outcome signals were converted through the image processor into binary images in such a manner that the animal was tracked as a black spot with a white background. The movement of the spot was recorded to track the position of the animals, the time they stayed in different zones and the distance traveled. The number of transitions between light and dark compartments in the LD test was evaluated on the video by an operator who was unaware of the group assignment of the mice.

The experiments were performed on each group of animals in the following order: LD choice test on day one (8th day of drug administration) and OF exploration test on the following day (9th day of drug administration). Mice were tested individually in each test arena and the operators stayed outside the testing room during the recording of the experimental session. Just before testing, the animals were allowed to acclimate to the room inside their cages for 3 min after being moved from their usual housing area.

The LD test arena consisted of two interconnected compartments that differed in size and color: a white open square (30 cm × 30 cm) and a black covered compartment (30 cm × 15 cm). Both compartments had walls that were 25 cm high. A small opening (4 × 4 cm) allowed the mice to freely move from the illuminated to the dark chamber and vice versa. The white field was brightly lit at 200 lux and the mice were left to explore the space for a 5 min testing period.

The OF test arena consisted of a 50 × 50 cm wooden platform, painted black and enclosed by 25 cm high walls. White light (100 lux) was present in the room. The 10 min test began with the subject being placed inside the OF arena, in a corner near the walls. Using the video-tracking system, the arena was virtually divided to define a square central zone occupying 25% of the total area. The tendency to enter the central zone and to travel inside it, instead of running along the walls or staying in the corners, is considered to be a sign of a decrease in the anxiety induced by a novel environment (neophobia) and of an increase in exploratory attitude, typical of mice that consider the experimental setting to be familiar. Thigmotaxis is particularly apparent upon the first exposure to a novel space, and helps the animal to define the boundaries of an unfamiliar environment. In unconditioned behavior tests in which an animal is placed for the first time in an arena, it also reflects novelty induced anxiety, general activity, exploratory behavior and decision-making [12].

### 2.4. Statistics

In the first series of experiments, the data from five experiments performed on three treatment groups of mice, which received either the 5 CH dilution or diazepam or a control placebo (total of five experiments = 72 mice per treatment group) were pooled and analyzed. In the second series of experiments, the data from one experiment performed on three treatment groups of mice, which received either the 5 or the 7 CH dilution or the placebo (total = 16 mice per treatment group) and those from two experiments performed with four treatment groups of mice, which received either the 5 CH or the 7 CH or the 30 CH dilutions or the placebo (total = 32 mice per treatment group) were pooled and analyzed. Extreme behavioral responses were considered as outlier data and excluded when their value exceeded 2 standard deviations (SDs). This occurred in very few cases (see Results section). When the distribution of data was normal (data from OF tests), the groups were compared by two-way analysis of variance (ANOVA SPSS, version 11 for Windows, Chicago, IL), using the treatment group and the experiments as the factors. The latter factor was included in the ANOVA analysis because the control values of activity varied considerably between experiments. Post-hoc *t*-tests were performed assuming equal variances with least significant difference (LSD) corrections to adjust for multiple comparisons. When the distribution was not normal (data from LD tests), the groups were compared by the non-parametric Kruskal-Wallis analysis of variance test, followed by the Mann–Whitney test for two independent samples to determine whether the activities of the control and drug-treated groups differed from each other.

The effect of the drugs on the various behavioral parameters was also calculated as a percentage with respect to the control values (taken as 100%) for each experiment, according to the formula:

Activity of each drug-treated animal/mean activity of control animals × 100.

This allowed the effects of the various dilutions tested in the two series of experiments, standardized as a percentage of their internal control values for untreated animals, to be compared and statistically evaluated.

## 3. Results

### 3.1. LD Choice

Table 1 reports the mouse behavior data in the LD test, in terms of the two main parameters: percentage of time spent in the illuminated area and the number of transitions between the two compartments. The mean time spent by control (placebo-treated) animals in the illuminated area was 15.98%, for mice treated with *G. sempervirens* 5 CH it was 18.11% (non-significant), while diazepam instead significantly increased the mean time spent in the illuminated compartment to 19.91% (*P* = .047). The number of transitions between the two compartments increased little in the *G*. *sempervirens* 5 CH-treated group, while in the diazepam-treated group the values rose from a mean of 6.19 (control) to a mean of 9.64 (*P* < .001).

### 3.2. OF Exploration

Table 2 reports the results for the OF tests. The placebo-treated control animals spent 6.34% of the time in the center of the OF arena. This value is considerably <25% one would expect in the case of a random choice, indicating that the central zone was quite uncomfortable for the mice. In the *G*. *sempervirens* 5 CH-treated group, the time spent in the central zone of the arena increased to 7.86% (*P* = .009), while the action of diazepam in this test was totally ineffective. The distance traveled by mice in the central zone of the arena was also positively influenced by treatment with *G*. *sempervirens* 5 CH, with an overall mean increase from 589.9 to 738.9 cm (*P* = .003). During the OF test the total distance traveled by the mice in the entire arena was also analyzed. No significant effect was found in drug-treated versus placebo-treated animals, indicating that the observed differences in time and distance spent in the central zone were not due to changes in the general, unspecific, locomotor activity of the mice.

The weight of the animals before and after the treatment was similar in the three treatment groups, suggesting no differences in feeding, metabolism or growth attributable to drug effects (data not shown). There was found to be a significant difference between the mean values for the five experiments (see notes to Tables 1 and 2), which was taken into account to control for its possible confounding effects. Finally, no significant interactions between groups and experiments were noted.

### 3.3. Study of a Different Mice Strain

We also performed one experiment in which the *G. sempervirens* 5 CH dilution/dynamization was tested on a different strain of mouse, C57BL. This yielded results qualitatively similar to those for the CD1 mice in both test models, confirming an increase in the time spent and distance traveled in the center of the OF induced by *G. sempervirens* 5 CH. However, due to the variability of responses between animals, the differences between control- and *G. sempervirens*-treated groups (e.g., 6.11 ± 0.80 and 8.83 ± 1.38% time spent in the center, respectively) in this individual experiment were not statistically significant (*P* = .068).

### 3.4. Even High Dilutions Work Better than Placebo

Following these positive results obtained with the 5 CH dilution/dynamization, we performed three further experiments, using the same protocol to also test higher dilutions, namely *G. sempervirens* 7 and 30 CH, in addition to 5 CH and a control placebo. Diazepam was not included here to avoid needlessly sacrificing animals, since the results for this control drug were sufficiently clear from the first series of experiments. The 5 and 7 CH dilutions/dynamizations of *G*. *sempervirens* were ineffective in the LD test; the 30 CH dilution slightly increased the values of the LD parameters, especially the number of transitions, but these effects were not statistically significant (data not shown). In the OF test (Table 3), the percentage time spent in the center of the arena and the distance traveled in the center were increased by all the *G*. *sempervirens* dilutions/dynamizations, with highly statistically significant results using the 7 and the 30 CH dilutions/dynamizations (*P* < .01). The effect of the 5 CH dilution/dynamization was quantitatively similar to that previously described in Table 2, but in these experiments it did not reach the statistical significance threshold, due to the lower size of subject population and to high inter-subject data variability (SEM). The maximum and most significant effect was obtained in the OF (distance traveled in the center) with the 30 CH dilution/dynamization (*P* = .002). In this series of experiments, too, the tested dilutions were found to have no significant effect on the unspecific locomotor activity, as indicated by the total distance traveled in the arena.

### 3.5. Summary of All the Experimental Data

**Figure 1:**
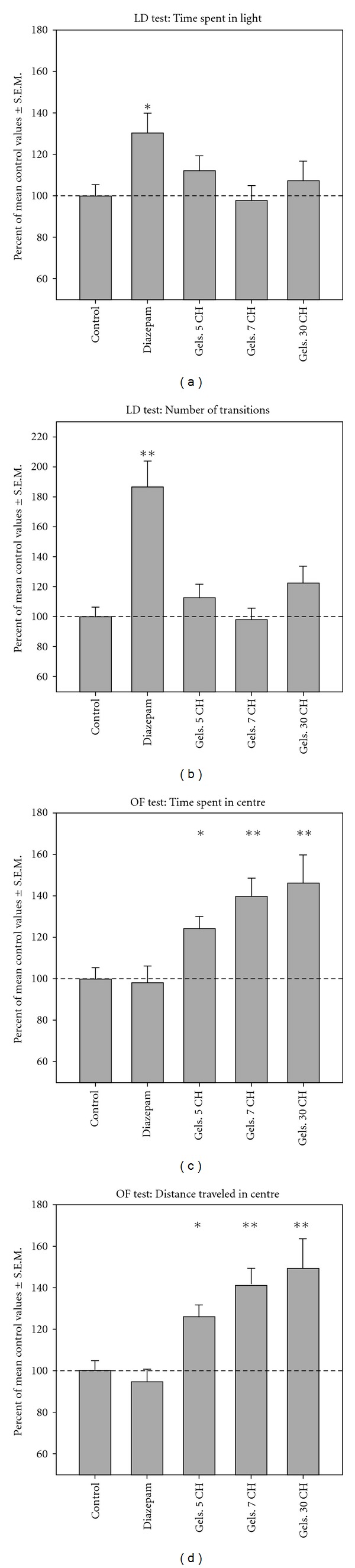
Summary of the effects of *G*. *sempervirens* 5, 7 and 30 CH, and of diazepam 1 mg/Kg bw on the main parameters of the LD test (Panels a and b) and of the OF test (Panels c and d), expressed as percent of the placebo-treated control values. In each experiment the functional activity of mice was calculated as a percentage with reference to the mean value of the placebo group (control vehicle). The values from eight experiments (control, placebo and 5 CH), from five experiments (diazepam), from three experiments (7 CH) and from two experiments (30 CH) were pooled and analyzed by two-way ANOVA or by the Mann-Whitney test as described in Section 1. *P < .01, **P < .001.

A summary of the main results of this investigation is given in Figure 1. The mean percentage values for all the mice of the treated groups confirms the increase due to diazepam but not due to *G*. *sempervirens* dilutions in the LD paradigm (panels (a) and (b)) and the significant increase due to all the tested homeopathic dilutions/dynamizations of *G*. *sempervirens* in the OF-tested variables (panels (c) and (d)).

## 4. Discussion

In the field of psychopathology, animal models have become an invaluable tool for analyzing the mechanisms of various disorders, and have aided in developing and predicting therapeutic responses to pharmacological agents such as benzodiazepines. In this work we investigated the effects of *G s*. *sempervirens* on the non-conditioned behavior of mice, using two widely validated models that allow the expression of various “symptoms” of emotional responses. We used ethological models in which the animal is influenced by emotional states of fear, curiosity or anxiety induced under different test paradigms without further conditioning, thus allowing for comprehensive “behavioral profiling”. Traditional difficulties in accepting these models stem from the argument that there is no conclusive evidence that what occurs in animals is equivalent to what occurs in humans. On the other hand, most conventional drugs and, recently, several homeopathic medicines have been tested in animal models, whose main advantages are that they allow multiple testing under controlled conditions and easier access to studying the mechanism of drug(s) activity.

### 4.1. Different Symptoms Modulation According to the Model

Most behavioral procedures for studying the pharmacology of anxiety use models involving non-conditioned behavior, that are usually based on novelty induced variations in exploratory activity. Considering the LD model, diazepam was active, as expected, significantly increasing the time spent in the light arena and the number of transitions. In the same assays, *G*. *sempervirens* was much less active than the conventional anxiolytic drug. Our data, showing small, but not significant, effects of *G*. *sempervirens* in the LD model, are in agreement with those of Bousta et al. [11] who reported some anxiolytic-like effects in mice stressed by repeated electric shock, but no effects in normal mice. Differences between the type and severity of external stressors or the animal strain or in the experimental setup might account for the high variability of results reported in different experimental conditions and by different laboratories [21, 22]. It has been noted that the extent to which an anxiolytic compound can facilitate exploratory activity depends on its baseline level in the control group [23]. Since our experimental setting did not involve prior exposure to stress, it is conceivable that in those conditions the response of the mice to *G*. *sempervirens* was slight because the level of basal anxiety, assessed by LD choice, was low.

In the OF model, all dilutions/dynamizations of *G. sempervirens* changed the behavior of the mice, reducing thigmotaxis, an anxiety-like behavioral response. The effects of *G. sempervirens* on the tendency of animals to stay and move in the center of the OF arena rather than in the periphery was consistent in the different experiments and highly statistically significant. Thigmotaxis is a very conspicuous behavior in anxious animals, and plays a role in the formation of avoidance behavior and cognition. It is therefore a primordial behavior, with a genetic basis, that is ecologically important and used by both animals and humans for exploring the environment.

The finding of a drug-induced increase in the distance traveled in the center of the arena, but not in the total distance traveled, suggests that the effects of *G*. *sempervirens* in our model system are anxiolytic rather than sedative or otherwise affecting animal locomotion. Since we used a control vehicle as a placebo in all experiments, the effects cannot be attributed to the very low dose of ethanol present in the final operating solutions, unlike in certain previous studies where the use of alcohol as vehicle for homeopathic preparations might have been able to produce some background effects in behavioral tests [24]. A further methodological issue that reinforces our findings is the systematic use of blinding, a procedure rarely employed in animal research, but which is worthwhile especially when testing hypotheses that are apparently in contrast with conventional scientific wisdom, such as the very existence of biological activity of solutions diluted beyond the Avogadro limit.

Since the reference benzodiazepine drug was inactive in the OF test, the results indicate that, at least in our experimental conditions, OF and LD explore different emotional responses, with different sensitivity to drugs and neurological mechanisms. This suggestion is in agreement with reports showing that anxiolytic treatments do not by themselves increase exploration in the central zone of the OF, but that they do decrease the stress-induced inhibition of exploration behavior [14]. It has been previously reported that the index of thigmotaxis, assessed by the OF test in CD1 mice, was not decreased by diazepam (≤5 mg/kg) [25]. Others reported anxiolytic effects of diazepam in the OF paradigm in rat [26, 27], but in mice 1 mg/kg [16, 28] or 1.5 mg/kg [29] diazepam did not show significant effects on the time spent in central area of OF. To the best of our knowledge open field behaviors of mice were reduced by diazepam at doses which may be within the sedative–hypnotic range [16, 28]. These conflicting drives could be influenced in various ways by drugs and small differences in the experimental conditions may have marked effects on the outcomes. Ethological models present individual differences and variable behavioral baseline levels and this requires strong care for variable parameters linked to environment, handling and testing [14].

### 4.2. Doses and Dilutions

Due to the complexity of the experimental setting, and in particular the need to use many animals in each group, in this study we started with a single dose (potency) of *G. sempervirens*, namely 5 CH, which was initially chosen because its dilution is sufficiently high to be representative of the homeopathic formulations commonly used in humans, but also sufficiently low to contain at least a few molecules of the purported active principle(s). We made the assumption that, given a theoretical concentration of the active principle in the 5 CH solution of 6.5 × 10^−14^ moles/l, its activity on the behavior of mice could be consistent with a molecular paradigm and with the existence of highly sensitive receptors for this drug in the animals' central nervous system, in agreement with other recent evidence [19, 20].

However, the results of the second series of experiments indicate that the drug may be active also at ultra-high dilutions/dynamizations. From our data (Figure 1) the 30 CH dilution/dynamization appears to be the most active in the OF variables, and these apparently paradoxical results require further confirmation. Indeed, at 30 CH, there is no active principle in this dilution/dynamization and further basic evidence will be necessary to make this observation acceptable. There is growing evidence in the literature that rodents are responsive to very high dilutions/dynamizations in immunological [30, 31] and behavioral [11, 32] models. If confirmed, these effects on mouse behavior would be much more coherent with the homeopathic paradigm and with traditional homeopathic medicine than initially expected.

A number of observations—coming from several research fields [11, 24, 31–34]—suggest that biologically active compounds may indeed have high-dilution effects which mimics those of lower dilutions (higher doses): in homeopathy there does not exist linearity or proportionality between molecular concentration of active principles and therapeutic effect [33–41].

So far there is no satisfactory or uniting theoretical explanation for these observations, but recent evidence seems to point to organization of the solvent water on a mesoscopic scale: the nano-heterogenous structure of water can be determined by interactive phenomena such as coherence [42–44], epitaxy [45, 46], temperature-pressure processes during strong agitation and formation of colloidal nanobubbles containing gaseous inclusions of oxygen, nitrogen, carbon dioxide, silica and possibly the remedy source material [46–51]. These unusual properties of high dilutions, which merit further investigation, are potentially relevant not just to homeopathic pharmaceutical practice, but also to basic research into cell sensitivity to regulation.

### 4.3. Hypothetical Mechanisms

**Figure 2:**
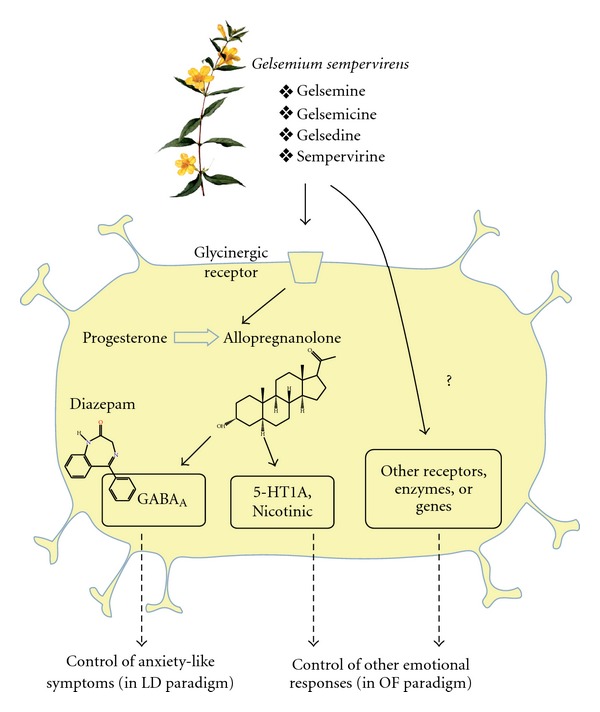
Hypothetical mechanism of action of *G*. *sempervirens*. Benzodiazepines act by enhancing the inhibitory effects of the neurotransmitter GABA. The neurosteroid allopregnanolone is produced from progesterone and acts both on the GABA receptor—at a binding site different from benzodiazepines—and on other receptors including the serotonine (5HT) and nicotinic-acetylcholine receptor, thus reducing the impulse generation in postsynaptic neurons. By fine tuning inhibitory transmission through glycinergic system and allopregnanolone synthesis [19, 20], *G*. *sempervirens* 5 CH may help the nervous system adapt to adverse situations. Due to the multicomponent nature of *G*. *sempervirens* and to our finding of preferential effects on OF paradigm even at high dilutions/dynamizations, the existence of other neurosteroid-independent mechanisms may be hypothesized.

A hypothetical diagram showing the possible action mechanism(s) of *G*. *sempervirens* is reported in Figure 2. A possible target of *G*. *sempervirens* action at neurological level has been identified by recent studies [19, 20], showing that in rat central nervous system (spinal cord and hippocampus) extremely low doses of this compound (10^−10^ M) and of its active principle gelsemine enhance the enzymatic production of the neurosteroid allopregnanolone (5a,3a-tetrahydroprogesterone), an active stimulator of GABAa and 5HT receptors and therefore of inhibitory signaling in the central nervous system. This effect could be due to a specific interaction at the level of the glycinergic receptors since this was antagonized by strychnine. However, since we noted differences in the action of conventional and homeopathic anxiolytic-like effects, it is highly conceivable that the multicomponent nature of the active principles of *G*. *sempervirens* make this compound able to interact with further receptor or gene expression systems, which in turn modulate the behavior in novel environments at more subtle and complex levels.

Beneficial, therapeutical effects of extremely low doses of agents which are toxic at high doses are defined hormesis. Recently Calabrese provided a broad range of examples of neurobiological processes and behavioral effects, including anxiety models, that exhibit non-linear, biphasic (“reverse-U”) dose responses to drugs and stressors [52, 53]. According to some authors, hormesis is not directly related to classical homeopathy [54], but it may represent one of the major explanations of the mechanism of action of homeopathic medicines when utilized in the molecular range of dilutions/dynamizations (approximately <9 CH) [45, 55, 56]. The development of interventions that activate hormetic signaling pathways in neurons is a promising new approach for the prevention and treatment of a range of neurological disorders [57].

## 5. Conclusions

Taken together, our data suggest that the effects of *G*. *sempervirens* at homeopathic dilutions on unconditioned mouse behavioral responses concern the exploratory attitude of the animals, a parameter that is better brought out by the OF test. Mouse behavior in the OF test is affected by various factors such as individual testing (since mice are social animals), neophobia (emotional response to a novel environment) and agoraphobia (being in an exposed setting from which there is no easy means of escape). In our experimental conditions *G*. *sempervirens* showed positive effects on these parameters, and the effect observed in the OF test fits very well with the classical archetype of the *G*. *sempervirens*. *Materia Medica*, according to which individuals who respond to this medicine are characterized by experiencing strong discomfort when confronted with novelty or unfamiliar situations.

In conclusion, homeopathic doses of *G*. *sempervirens* positively influence the emotional responses of mice to novel environments, suggesting an improvement in exploratory behavior and a decrease in thigmotaxis or neophobia. The study provides a basic knowledge of the pharmacological effects of *G*. *sempervirens* and can implement the clinical research on the anxiolytic effect of this drug on humans, which are based only on empirical knowledge but not on fundamental evidence supplied by controlled investigations.

## Funding

Grants from Laboratoires Boiron s.r.l. (Milan, Italy) to Verona University and from the Ministry of University and Scientific and Technologic Research.

## Notation

Recently the same group showed that following small methodological changes a significant anxiolytic-like effect of Gelsemium s. is detected also in the Light-dark test (Magnani P, Conforti A, Zanolin E, Marzotto M, Bellavite P. Dose-effect study of Gelsemium sempervirens in high dilutions on anxiety-related responses in mice. Psychopharmacology 210(4):533-545, 2010).

## Figures and Tables

**Table 1:** Effects of *G. sempervirens* 5 CH and diazepam on the behavioral parameters in the LD.

Parameter	Control			*G. sempervirens* (5 CH)				Diazepam			
	*n*	Mean	SEM	*n*	Mean	SEM	*P* ^a^	*n*	Mean	SEM	*P* ^a^
Percentage of time in light*	71	15.98	1.29	69	18.11	1.42	.341	69	19.91	1.39	.047
Number of transitions**	71	6.19	0.58	70	6.40	0.56	.699	70	9.64	0.64	<.001

^a^Mann—Whitney test of treated group versus control.

*Kruskal Wallis for groups *P* = .159.

**Kruskal Wallis for groups *P* < .001.

**Table 2:** Effects of *G. sempervirens* 5 CH and diazepam on the behavioral parameters in the OF test.

Parameter	Control			*G. sempervirens* (5 CH)				Diazepam			
	*n*	Mean	SEM	*n*	Mean	SEM	*P* ^a^	*n*	Mean	SEM	*P* ^a^
Percentage of time in center*	69	6.34	0.41	70	7.86	0.41	.009	69	6.21	0.48	.812
Distance in center**	68	589.9	37.8	70	738.9	38.0	.003	68	585.1	49.5	.925
Total distance a***	70	5181.5	166.2	71	5371.4	168.1	.306	71	5321.7	211.1	.449

^a^Post-hoc analysis of treated group versus control.

Two-way ANOVA for groups: *P < .001, **P = .001, ***P = .647.

For experiments *P < .001, **P = .001, ***P < .001.

**Table 3:** Effects of *G. sempervirens* 5, 7 and 30 CH on the behavioral parameters in the OF test^a^.

Parameter	Control			*G. sempervirens* (5 CH)				*G. sempervirens* (7 CH)				*G. sempervirens* (30 CH)			
	*n*	Mean	SEM	*n*	Mean	SEM	*P* ^b^	*n*	Mean	SEM	*P* ^b^	*n*	Mean	SEM	*P* ^b^
Percentage of time in center*	47	5.98	0.51	48	7.09	0.61	.169	47	8.19	0.52	.007	32	8.60	0.80	.004
Distance in center**	45	501.1	42.6	48	618.5	46.6	.089	47	702.6	41.3	.004	32	744.0	75.1	.002
Total distance***	47	4892.9	167.6	47	5180.3	146.4	.183	46	5248.7	171.7	.102	31	5158.1	235.4	.378

^a^Data are pooled from three experiments (control, 5 CH and 7 CH) and from two experiments (30 CH).

^b^Post-hoc analysis of treated group versus control.

Two-way ANOVA for groups: *P = .012, **P = .008, ***P = .371. For experiments *P = .075, **P = .867, ***P < .001.
